# Tenecteplase vs. alteplase for acute ischemic stroke: a systematic review

**DOI:** 10.1186/s12245-021-00399-w

**Published:** 2022-01-04

**Authors:** Neha Potla, Latha Ganti

**Affiliations:** 1 Unionville-Chadds Ford School District, Kennett Square, PA USA; 2 Departments of Neurology and Emergency Medicine, University of Central Florida College of Medicine, Orlando, FL USA; 3 Envision Physician Services, Plantation, Florida, FL USA

## Abstract

**Introduction:**

Thrombolysis for acute ischemic stroke (AIS) with alteplase is the currently approved therapy for patients who present within 4.5 h of symptom onset and meet criteria. Recently, there has been interest in the thrombolytic tenecteplase, a modified version of alteplase, due to its lower cost, ease of administration, and studies reporting better outcomes when compared to alteplase.

This systematic review compares the efficacy of tenecteplase vs. alteplase with regard to three outcomes: (1) rate of symptomatic hemorrhage, (2) functional outcome at 90 days, and (3) reperfusion grade after thrombectomy to compare the efficacy of both thrombolytics in AIS

**Methods:**

The search was conducted in August 2021 in PubMed, filtered for randomized controlled trials, and studies in English. The main search term was “tenecteplase for acute stroke.”

**Results:**

A total of 6 randomized clinical trials including 1675 patients with AIS was included. No one’s study compared alteplase to tenecteplase with all three outcomes after acute ischemic stroke; however, by using a combination of the results, this systematic review summarizes whether tenecteplase outperforms alteplase.

**Conclusions:**

The available evidence suggests that tenecteplase appears to be a better thrombolytic agent for acute ischemic stroke when compared to alteplase.

## Introduction

Alteplase is the only the Food and Drug Administration (FDA) approved thrombolytic for thrombolysis for acute ischemic stroke (AIS). When given to eligible patients within 4.5 h, there is a 28% decrease in disability at 90 days, and a more rapid improvement is associated with greater symptom improvement [1]. The risk of symptomatic hemorrhage is 6% in all-comers [2]. Patients can be further risk stratified depending on the number of the following risk factors they possess: NIHSS>20, glucose > 300 mg/dL, age > 70 years, and ischemic changes on CT [3], making the risk range 1.8 to 21.2% [3]. For patients with a stroke mimic such as migraine or hypoglycemia, the risk is much lower. In a stroke mimic cohort of 107, there were zero instances of intracranial hemorrhage after administration of alteplase [4].

Both alteplase and tenecteplase are thrombolytic agents that achieve their effect by binding to fibrin in clots and converting entrapped plasminogen to plasmin. Plasmin in turn breaks up the thrombus. Tenecteplase is a modified form of alteplase with three point mutations that renders it a larger molecule with a longer half-life [5]. These properties enable it to be given as a single bolus. This is especially helpful when a patient requires transfer from a primary to a comprehensive stroke center, for example. Differences between the drugs are summarized in Table 1.

**Table 1:** Alteplase vs. Tenecteplase

	Alteplase	Tenecteplase
Fibrin selectivity	medium	high
Half-life	5 min	17 min
Dosing	bolus plus infusion	single bolus

Tenecteplase has been studied for over 20 years in the myocardial infarction (MI) population. When compared to alteplase for MI, tenecteplase showed equal vessel patency at 90 min [6] and equal mortality at 30 days [7]. Given the long-standing success tenecteplase has in treating MI, naturally, physician-scientists have hypothesized its application in AIS, and subsequent trials have emerged.

This systematic review focuses on the effects of tenecteplase compared to the effects of alteplase in treating patients with acute ischemic stroke. The objective of this review is to summarize the impact of both thrombolytics on (1) the rate of symptomatic hemorrhage, (2) the functional outcome of the patient after 90 days, and (3) the reperfusion grade after the thrombectomy.

## Materials and methods

Strategies used to select studies, extract data, and make objective assessments based on both qualitative and quantitative information from the available literature were performed according to the Preferred Reporting Items for Systematic Review (PRISMA) guideline [8]. A formal protocol for this review was not registered prospectively.

### Search strategy

Studies were independently searched PubMed using the following search terms: “tenecteplase for acute stroke.” The search was limited to human randomized controlled trials. Only studies written in English were searched. Bibliographies of retrieved articles were manually checked for additional references. Studies were included if they met any of the following criteria: administration of tenecteplase for AIS and any discussion of (1) occurrence of symptomatic hemorrhage, (2) functional outcome at 90 days, and (3) reperfusion grade after thrombectomy. Functional outcome was assessed using the modified Rankin score (mRS). Reperfusion grade was assessed using the thrombolysis in cerebral infarction (TICI) perfusion scale, graded from 0 (no perfusion) to 3 (complete perfusion).

The search was conducted in August 2021.

### Eligibility criteria

Studies were included if they met the following Population, Intervention, Controls, Outcome, Study (PICOS) criteria [9]:

1. Population: The study population included adults (18 years old or older) diagnosed with acute ischemic stroke confirmed by diagnostic guidelines updated by the American Heart Association/American Stroke Association [10].
2. Intervention: Interventions had to be treatment-based, including tenecteplase or alteplase intravenously administered.
3. Controls: Because the goal of systematic review is to compare the current medication utilized by physicians to a medicine not yet approved, there is no formal control. If anything, alteplase is the control of the studies because the goal was to assess if tenecteplase had a comparatively better, equal, or worse outcome.
4. Outcome: Outcome measures had to assess the occurrence of hemorrhage, functional outcome (modified Rankin Score measured at 90 days), and reperfusion grade after thrombectomy.
5. Study design: All designs had to be randomized controlled trials.
6. Time: Articles considered included those published between February 2010 and August 2021.

Studies were excluded from the review if they were not written in English and if the authors had a bias for or against a given drug. In addition, pulmonary embolism and myocardial infarction studies assessing the combined effects of both drugs were also excluded from the review.

### Study selection and data extraction

**Fig. 1:**
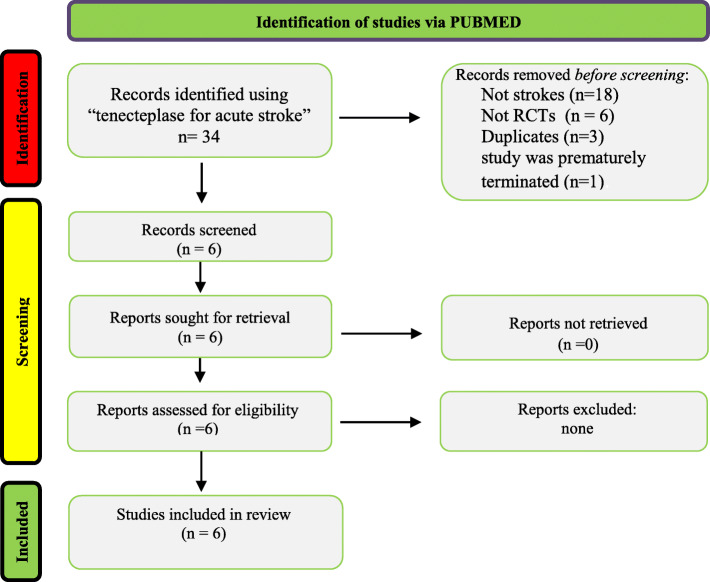
Flow diagram for inclusion of studies

We only included randomized control trials comparing the effects of the administration of tenecteplase (10–40 mg) to alteplase (90 mg) in patients with acute ischemic stroke. The primary efficacy endpoint analyzed was treated patients’ absolute risk of symptomatic intracranial hemorrhage (sICH) and functional ability at 3 months post-stroke, and their reperfusion grade if they underwent thrombectomy. Symptomatic hemorrhage events in trials were identified using the specific sICH definition clarified by that trial. Any discrepancies in the study selection were resolved by consensus. Full texts were retrieved and evaluated based on the previously set inclusion criteria. See Fig. 1.

### Data extraction and analysis

Data were extracted and documented based on predetermined criteria to identify solely relevant information. Details recorded from each reference include the author’s last name, study publication year, participants, intervention, outcomes measures, and results of the study. Data were extracted by one reviewer and checked for accuracy by the second reviewer. Each of the studies and patients’ characteristics and details of intervention is summarized in Table 1. The table depicts the similar nature of each study and the comparability of each of the studies’ outcomes.

**Fig. 2:**
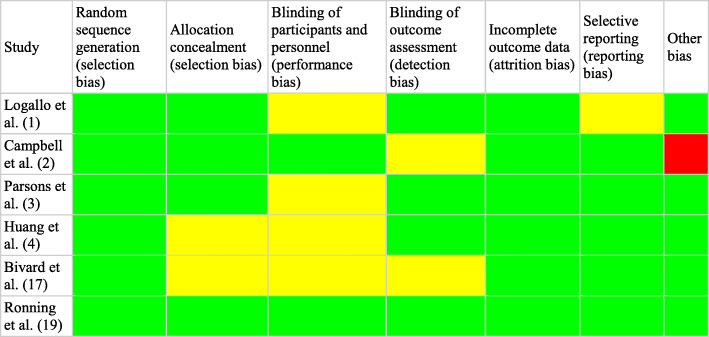
Risk of bias by individual study

**Fig. 3:**
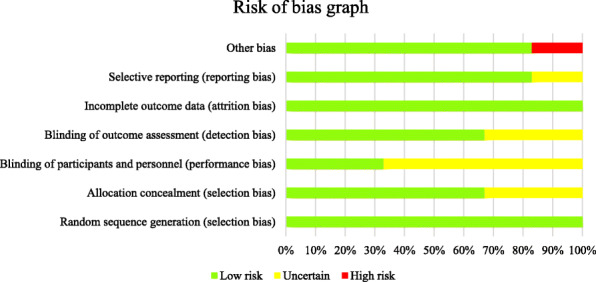
Risk of bias by percentage across all included studies

The risk of bias assessment was created based on the handbook of Cochrane (5.1 version) [11]. To assess bias, one reviewer independently followed steps to choose articles of relevancy; if the study’s main question was answered unclearly, we chose “unclear.” Reviewer selection, performance (blinding), detection (double-blinding), attrition, selective reporting, and other sources of bias were all factors included in the bias assessment (Figs. 2 and 3).

## Results

### Study characteristics

A total of 34 articles were retrieved in this search. Thirty-three articles were considered for this screening, and 28 articles were excluded because the study did not focus on stroke patients (*n* = 18), the type of study did not meet our inclusion criteria (*n* = 6), the article came up twice through the search (*n* = 3), or the study was prematurely terminated (*n* = 1). Six articles were included in the systematic review. See Fig. 1.

### Baselines of patients

In total, the data consisted of 1675 patients treated with intravenous thrombolytics for acute ischemic stroke. A total of 782 patients received tenecteplase while 727 received alteplase (not including 5 because of a lack of specification). Based on the available information, the mean age of the participants across 3 studies was 70.4 years (SD = 14.4) for patients using tenecteplase and 71.3 (SD = 15.9) for patients using alteplase, ranging from 49 to 92 years across all treatments [12–14]. All subjects suffered from acute ischemic stroke and were followed up for 90 days. The remaining studies either measured their data using the median or did not provide an age range. One study provided a median age of 71 years between both treatments (IQR = 64–79) [11]. The remaining 1 study did not provide age data [15].

### Risk of bias of included studies

Each of the studies’ randomization, allocation blinding, incomplete outcome data, double-blinding, and other sources of bias were assessed as a low risk of bias in most of the included studies [12, 13, 16]. Blinding of participants scored a high risk or unclear risk in 1 of the studies [15]. A letter to the article describes that the researchers committed selection bias for “small cerebral infarctions” and furthermore exaggerated the benefits of tenecteplase versus alteplase in patients with ischemic stroke [17]. There are some studies where the blinding outcomes were either not described or unclear so they were scored unclear in the detection of bias [14].

### Intervention characteristics

Study objectives did not vary for most of the studies: tenecteplase versus alteplase for acute ischemic stroke [12–16], tenecteplase’s effect on recanalization on patients with ischemic stroke [16], and most effective time frame to use tenecteplase/alteplase after ischemic stroke [16]. To determine the effectiveness with the current understanding that alteplase can help patients with AIS, tenecteplase was compared with essentially one control group: alteplase [12–16].

All six trials involved a randomized, prospective, open-label, and blinded endpoint trial with prior baseline CT scans to first establish lack of intracerebral hemorrhage [12–17]. In five of the trials, thrombolytics were administered within 4.5 h of symptom onset [12–14, 16] while in one trial it was within 6 h [15]. Following diagnosis, patients were randomized to the following treatment options: 0.4 mg/kg dose of tenecteplase v. 0.9 mg/kg dose of alteplase [12, 16] or 0.25 mg/kg dose of tenecteplase v. 0.9 mg/kg dose of alteplase [13, 14] or 0.1 mg/kg dose of tenecteplase v. 0.25 mg/kg dose of tenecteplase v. 0.9 mg/kg dose of alteplase [15]. To measure the degree of disability following the stroke treatment, the modified Rankin Score (mRS) was used as the main metric in five studies [12, 13, 15]. The mRSis a 6-point disability scale where 0 means no disability and 6 means dead.

Patients were not informed of treatment allocation. Within 24–48 h of treatment, symptomatic intracranial hemorrhage and intracranial hemorrhage were ascertained [12–16]. Other studies additionally tested for reperfusion rates within 24 to 48 h [13, 15].

### Qualitative synthesis: outcome

The included studies (Table 2) show that tenecteplase is either better or has an equivalent effect as alteplase on patients with AIS.

**Table 2:** Summary of outcomes of included studies

Outcome measures	Measurements	Results
Rate of symptomatic hemorrhage	Baseline and after-treatment variables with symptomatic and asymptomatic	Following treatment with tenecteplase, there was a greater early clinical improvement with a median of 9 in comparison to alteplase’s median of 1 [13].
	National Institutes of Health Stroke Scale score (NIHSS)	No significant difference between both scores because a majority of the score range fell between 0 and 4 for both interventions [16].
Functional outcome at 90 days	Modified Rankin Scale (mRS)	Both interventions shared the same effect [12, 16].
		A higher proportion of patients showed a significant recovery using the tenecteplase intervention [15].
		The proportion of patients with good functional outcome was 61% in the tenecteplase group and 57% in the alteplase group (odds ratio, 1.24; 95% CI 0.65–2.37).
Reperfusion rate after thrombectomy	Modified thrombolysis in cerebral infarction (mTICI)	Over the course of 90 days following the treatment, overall reperfusion rates were significantly higher than alteplase [13].
		Tenecteplase was associated with significantly better reperfusion (*P*=0.004) and clinical outcomes than alteplase (*P*<0.0001) [15].

### Rate of symptomatic hemorrhage

Four trials showed insignificant differences in the percentage of patients with symptomatic hemorrhage [12–14, 16]. All studies noted that there would need for additional tests to conclude that treatment with a certain dose of tenecteplase exposes patients to a higher risk of bleeding complications than alteplase does and vice versa. Logallo et al. note any hemorrhage occurred in 9% of patients taking tenecteplase and also 9% of patients taking alteplase (*P*=0.82) [12]. Campbell et al. reported intracranial hemorrhage rates were 15% for tenecteplase patients versus 29% for alteplase patients (*P*=0.091) [13]. It may be important to note that Logallo et al. utilized a 0.4 mg/kg dose of tenecteplase and Campbell et al. utilized a 0.25 mg/kg dose of tenecteplase. Huang et al. do not mention intracranial or symptomatic hemorrhage as one of their outcomes, therefore goes unmentioned during the review. Ronning et al. conclude that tenecteplase in fact had a significant effect in reducing symptomatic hemorrhage in comparison to alteplase. Specifically, intracerebral hemorrhages only impacted 2 of the 75 patients in the tenecteplase pool and 5 of the 71 patients in the alteplase (*P*=0.002) [16]. It is important to note that alteplase does not exceed the function or utility of tenecteplase; however, tenecteplase is either equivalent or better than the function of alteplase.

### Functional outcome at 90 days

Two studies reported on functional outcome, and there was no statistically significant difference between tenecteplase and alteplase. Logallo et al. identified an excellent functional outcome for 64% of the patients in the tenecteplase pool and 64% of the patients in the alteplase pool (*p*=.98) [12]. Roning et al. show insignificance as well with 57% of patients that received tenecteplase and 53% of patients that received alteplase attained a good functional outcome (mRS 0-1) at 90 days [16].

### Reperfusion rate after thrombectomy

In three trials, the criteria of “reperfusion rates after thrombectomy” was not mentioned, quantified, or the focus of their papers [12, 14, 16]. In the remaining three studies, tenecteplase was superior at increasing the reperfusion rate after thrombectomy. Campbell et al. reported 22% of the patients in the tenecteplase group and 10% of the patients in the alteplase group saw an increase in blood flow in the formerly blocked artery (*P*=0.002) [13]. Parsons et al. report reperfusion rates were significantly better for patients treated with tenecteplase (*P*=0.004) [15]. Ronning et al. confirmed these findings by concluding that tenecteplase increased recanalization better than alteplase [16].

## Discussion

Acute ischemic stroke remains the leading cause of disability worldwide and confers a significant burden to the quality of life for the stroke survivor and their caregivers. Because of this, there is tremendous interest in both targeted and supportive therapeutics to help ease this burden. Research has been done on the impact of acute blood pressure [18–20], anticoagulants [21], corticosteroids [22], and even antibiotics for AIS [23]. Thrombectomy is an excellent option for large vessel occlusions (LVOs), but for non-LVOs, thrombolytics remain the mainstay of treatment. As such, there is a great of interest in finding the best options within the thrombolytic class and that is where this systematic review enriches the existing literature.

Tenecteplase also has additional benefits. It can be given as a single bolus, which is more comfortable for the patient, less arduous for the hospital staff, and certainly more convenient for prehospital or interhospital transfer. Tenecteplase is also currently cheaper. A 50 mg vial of tenecteplase costs approximately $6300 while a 100 mg vial of alteplase costs approximately $9200 [24].

## Conclusion

The overarching purpose of this systematic review was to evaluate the effectiveness of tenecteplase versus alteplase on AIS patients eligible for thrombolytic therapy. The results demonstrate that tenecteplase is just as good and in some cases better than alteplase with regard to the outcomes of (1) post thrombolytic bleeding, (2) functional outcome at 90 days as measured by the mRs, and (3) recanalization/reperfusion rates following thrombectomy.

## Data Availability

All data generated or analyzed during this study are included in this published article and its supplementary information files.

## Abbreviations

AIS: Acute ischemic stroke
FDA: Food and Drug Administration
NIHSS: National Institutes of Health Stroke Scale
CT: Computed tomography
MI: Myocardial infarction
PRISMA: Preferred Reporting Items for Systematic Reviews
mRS: Modified Rankin Score
LVO: Large vessel occlusion
